# Real-time detection of singlet-oxygen signatures using a single-photon avalanche diode detector

**DOI:** 10.1364/BOE.568337

**Published:** 2025-07-07

**Authors:** Arran Sykes, Lisa Saalbach, Sam Benson, Eleni Nestoros, Rachael Tobin, Xin Yi, Michael G. Tanner, Marc Vendrell, Gerald S. Buller

**Affiliations:** 1 Institute of Photonics and Quantum Sciences, Heriot-Watt University, EH14 4AS Edinburgh, United Kingdom; 2 Centre for Inflammation Research, The University of Edinburgh, Edinburgh BioQuarter, 4-5 Little France Drive, EH16 4UU Edinburgh, United Kingdom; 3 IRR Chemistry Hub, Institute for Regeneration and Repair, The University of Edinburgh, EH16 4UU Edinburgh, UK

## Abstract

Singlet-oxygen, the first excited state of molecular oxygen (O_2_), is a reactive oxygen species that plays a key role as a cytotoxic agent in photodynamic therapy (PDT). In this work, we report a highly light-sensitive detection system based on a single-photon avalanche diode (SPAD) detector and time-correlated single-photon counting (TCPSC) for real-time detection of luminescence signatures from photosensitized singlet-oxygen. Dynamics of singlet-oxygen produced by the excitation of small-scale organic nitrobenzoselenadiazole photosensitizers were extracted with acquisition times as short as 1 second. In a clinical setting, the ability to detect singlet-oxygen production in short time frames could allow for real-time adjustments in light dosing, ensuring sufficient singlet-oxygen production and complete treatment of diseased tissue in applications such as PDT.

## 1. Introduction

The production of singlet-oxygen, a highly reactive oxygen species and the first excited state of molecular oxygen, plays an important role in various photochemical and photobiological processes [1]. Singlet-oxygen is cytotoxic in nature [2], therefore enabling its use to eradicate cancerous tissue or anti-biotic resistant bacteria via numerous routes depending on its localization and concentration within a cell. This can include programmed or unprogrammed cell death pathways, mitochondrial or cell membrane/wall lysis as well as damage to the tumor vasculature [3–5]. By purposefully invoking these processes singlet-oxygen has been used in blood sterilization [6], wastewater treatment [7], insecticides and herbicides [8], photodynamic therapy (PDT) for cancer [9–11] and antimicrobial PDT [12–14]. In PDT, photosensitizers are used as part of a pathway to excite oxygen molecules in vivo, creating the excited singlet-oxygen state, which can selectively destroy lesions or tumors.

**Fig. 1.**
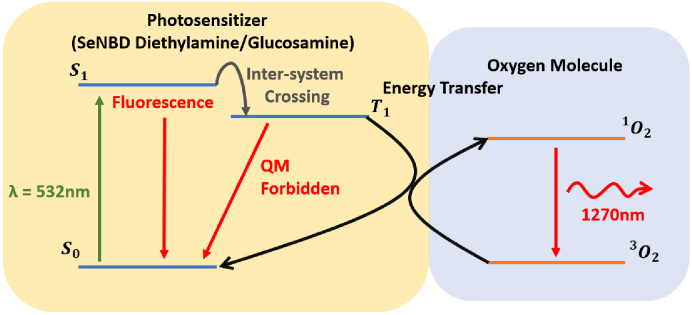
A Jablonski diagram of the relevant energy transfer processes for the singlet-oxygen production via the interaction between a photosensitizer and oxygen molecule.

Figure 1 outlines the photosensitized production of singlet-oxygen. The photosensitizer is excited to its first electronic excited state ( $S_{1}$ ) and, subsequently, undergoes intersystem crossing to a lower-lying triplet state ( $T_{1}$ ). Energy transfer between this photosensitizer triplet state and molecular oxygen results in the excitation of oxygen to its O2(Δg1) state. Relaxation from this state to the electronic ground state of molecular oxygen leads to the emission of a photon at ∼
 1270 nm which can be detected to monitor the presence of singlet-oxygen. Singlet-oxygen production is heavily influenced by photosensitizer concentration and oxygen concentration in its surrounding environment [15,16]. The time-dependence of the singlet-oxygen concentration, [Og1] can be described by:

(1) [Og1]=Nσ
[S0]ϕ
Dτ
Dτ
T−
τ
D(e−
tτ
T−
e−
tτ
D),

where N is the number of incident photons, σ
 is the absorption cross-section of the photosensitizer, $\left[{\text{S}}_{0}\right]$ is the concentration of the photosensitizer ground state and ϕ
D is the quantum yield of singlet-oxygen. τ
T is the signal rise time typically linked to the lifetime of the photosensitizer triplet state and τ
D is attributed to the lifetime of singlet-oxygen [17,18].

Photomultiplier tubes (PMTs), which typically have a large collection area but require high operation voltages, were initially the primary detector for investigating singlet-oxygen signals [19–22]. Recently, superconducting nanowire single-photon detectors (SNSPDs) with very high detection efficiencies [23,24] have been used to detect phosphorescence from photosensitized singlet-oxygen but require operation temperatures below 10 K. Single-photon avalanche diodes (SPADs) are an alternative technology to detect single photons at short-wave infrared wavelengths [18,25–27]. Recently, a free-running InGaAs/InP SPAD was used to investigate the singlet-oxygen production via the photosensitizer Protoporphyrin IX in varying concentrations [27] and in Rose Bengal studying the effects of scattering medium on lifetimes [28]. Real-time monitoring of singlet-oxygen phosphorescence has been carried out using spectroscopic methods, such as luminescence microspectroscopy in combination with an InGaAs-based camera [29]. Similarly, the singlet-oxygen quantum yield was monitored in real-time using time-resolved spectroscopy by Liu *et al.* [30]. Multispectral singlet oxygen luminescence dosimetry (MSOLD) also allows for real-time dosing by spectrally separating the PS luminescence and the SO signal [31,32]. Continuous SOLD (CSOLD) by Lu *et al.* is a similar method verified alongside MSOLD [33]. Such real-time monitoring using spectroscopy methods typically requires more complex experimental systems and data analysis compared to the time-correlated single-photon counting (TCSPC) based method for real-time singlet-oxygen signature detection proposed here.

In this work, singlet-oxygen was produced using small-molecule organic nitrobenzoselenadiazole (SeNBD) based photosensitizers [34,35] in DMSO. These small organic SeNBD-based photosensitizers can target specific cells based on their metabolic structures, selectively killing these cells with high spatial resolution [36]. Singlet-oxygen signatures from the photosensitizer and solvent combinations were monitored using an efficient detection system based on a free-running InGaAs/InP SPAD in conjunction with the time-correlated single photon counting (TCSPC) technique. Singlet-oxygen and photosensitizer triplet state lifetimes were extracted from TCSPC measurements with acquisition times as short as 1 second - demonstrating the possibility of singlet-oxygen signature detection in real-time using a SPAD detector-based system.

## 2. Method

### 2.1. Materials

Nitrobenzoselenadiazole (SeNBD) Diethylamine (SeNBD Die) and SeNBD Glucosamine (SeNBD Glu) [34,35] were dissolved in analytical grade dimethylsulfoxide (DMSO) at a fixed concentration of 50 μ
 M. Three unique samples of each photosensitizer in DMSO were measured. The photosensitizer showed an absorption peak centred at 510 nm. For further detailed information regarding the photosensitizers we refer to the work by Benson *et al.* [35]. The photosensitizers were highly soluble in DMSO. When dissolved in water they showed no singlet-oxygen production as previously observed [35]. Rose Bengal in water, a well-studied photosensitizer molecule [18,23–25], again at a concentration of 50 μ
 M, was used to benchmark the detection system.

### 2.2. System

**Fig. 2.**
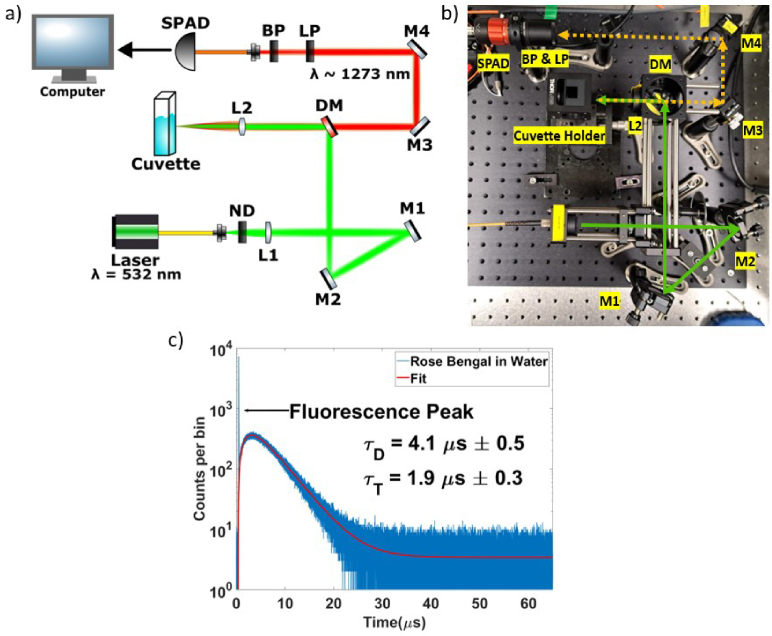
(a) A schematic of the singlet-oxygen luminescence detection system. A 532 nm wavelength laser is focused onto the sample using lens L2 ( f = 75 mm). A dichroic mirror (DM) reflects the excitation beam towards the sample and transmits the longer wavelength singlet-oxygen phosphorescence emission towards the detection channel. A longpass filter (LP) and a bandpass filter (BP) remove unwanted signal wavelengths prior to detection at the InGaAs/InP SPAD. b) An annotated photo of the set-up. c) Timing histogram (timing bin size of 1.024 ns) of the luminescence collected from excitation of Rose Bengal in water measured over a 30-minute acquisition time and fitted using Eq. (1).

Figure 2(a) illustrates the experimental setup used for the excitation of the photosensitizer samples and subsequent detection of singlet-oxygen luminescence. The illumination source is a fibre-coupled Q-switched laser emitting at a wavelength of 532 nm (Crystalaser QL-532-500), operated at a 15 kHz repetition rate and a pulse width of 8 ns. The repetition rate determines the temporal window observable using this TCSPC-based method. A 15 kHz repetition rate results in a maximum temporal window of 66.7 μ
 s. The emitted laser power is adjusted using a variable neutral density (ND) filter to allow for power-level optimization of the beam used to excite the sample. The beam is collimated by lens L1 ( f = 75 mm) and two steering mirrors direct the light towards a dichroic mirror (DM), which reflects the 532 nm wavelength excitation beam towards lens L2 ( f = 75 mm), used to focus the beam onto the sample. The laser power at the sample was sample-dependent and ranged between 0.5 mW and 1.6 mW with a focused beam diameter of 400 μ
 m at the sample. For clinical applications, it is critical to maintain low-power optical illumination to avoid photo-structural damage to healthy cells. Employing a low-power laser source additionally reduces cost and provides improved eye-safety. The luminescence emitted by the excited sample is collected and passed back through the DM towards the detector collection optics assembly consisting of a longpass filter (cut-on λ
 = 1000 nm), a bandpass filter (central λ
 = 1275 nm with a full width at half maximum of 50 nm). A large, clear aperture fibre collection package was used to couple the light into the InGaAs/InP SPAD detector. The detector used is an ID Quantique ID230 free-running InGaAs/InP SPAD featuring a very low dark count rate (as low as < 50 cps [37]). The detector was run at ${\mathrm{-90}}^{\circ }$ C with 20% detection efficiency, 100 μ
 s dead-time (to minimize afterpulsing) and the average detector count rate was limited to about 5% of the laser repetition rate ( ∼
750 cps) to avoid the deleterious effects of pulse pile-up which can significantly affect the estimated lifetime. A 15 kHz timing synchronization signal from the laser is used to trigger a HydraHarp400 TCSPC module, allowing for the construction of a histogram of the number of photons against arrival time for the luminescence collected from the samples. Rise- and fall-times of the signal can then be extracted. Average background count rates (including detector dark counts) were in the range of 80-120 cps and were subtracted prior to data fitting. For each photosensitizer and solvent sample, an acquisition time of 30 minutes was used to define accurate lifetime values for comparison with lifetime values extracted at much shorter acquisition times. The experimental system was initially characterized using Rose Bengal in water. As the system is time-resolved, an established photosensitizer and solvent combination can be used to verify that the detected signal originates from singlet-oxygen, via the singlet-oxygen lifetime. A TCSPC histogram obtained for a 30-minute acquisition time measurement of Rose Bengal in water is shown in Fig. 2(c). Equation 1 is fitted to the histograms to extract values for the singlet-oxygen lifetime, τ
D and the triplet-state lifetime τ
t . The first few time bins containing the early fluorescence signal (a signature from the photosensitizer) are removed before fitting for all samples. A singlet-oxygen lifetime, τ
D of 4.1 μ
 s and triplet state lifetime, τ
t , of 1.9 μ
 s were found for Rose Bengal in water, which is in good agreement with previously reported values obtained using SPAD-based detection systems [18,28], validating that the detected luminescence signal originates from photosensitized singlet-oxygen.

## 3. Results and discussion

**Fig. 3.**
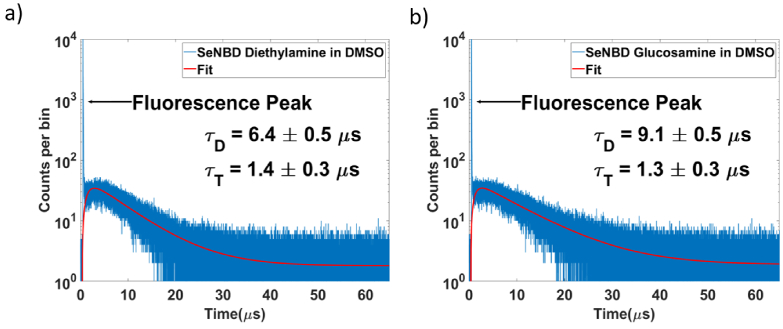
TCSPC histograms (timing bin size of 1.024 ns) showing the phosphorescence signal associated with singlet-oxygen production from SeNBD Die and SeNBD Glu in DMSO (a) and (b), respectively. A sharp initial fluorescence peak is present but discounted for data fitting using Eq. (1). Singlet-oxygen τ
D and triplet state τ
T lifetimes extracted from the fitting of the decay and rise-time of the signal in each case.

Figure 3 shows TCSPC histograms, and corresponding fits, for SeNBD Die (a) and SeNBD Glu (b) in DMSO acquired over a measurement time of 30-minutes. Similar to the histogram shown earlier for Rose Bengal in water (Fig. 2 c) a sharp fluorescence signal is seen in early time bins (up to ∼
 700 ns) of the histograms. This is followed by the longer-lived phosphorescence signal indicating the presence of singlet-oxygen. As previously, the time bins containing the fluorescence peak are removed before fitting Eq. (1) to the phosphorescence signal, which yields the singlet-oxygen ( τ
D ) and photosensitizer triplet-state ( τ
T ) lifetimes. These dynamics provide information on the interaction between the photosensitizer and its environment, any quenching of the fluorophore and the relative stability of the excited states. At 30-minute acquisition times, SeNBD Die and SeNBD Glu in DMSO exhibit τ
D lifetimes of τ
D = 6.4 μ
 s and 9.1 μ
 s, respectively. These observations of the dynamics of SeNBD Die and SeNBD Glu in two solvents align with previous work, which showed that SeNBD Glu and SeNBD Die are environmentally sensitive [36]. Regarding the variation of τ
D in SeNBD Die and SeNBD Glu in DMSO, we note that there is a significant spread in τ
D values reported in the literature for various photosensitizers in DMSO which range from 19 μ
s [38], over 5.5 μ
s [39,40], to as low as 1.8 μ
s [41]. This variation suggests potential photosensitizer and solvent interactions, due to the different attached groups in the photosensitizers, are likely affecting the singlet-oxygen lifetimes. The extraction of the photosensitizer triplet state lifetime, τ
T from the TCSPC histograms is more challenging as the rise of the phosphorescence signal can be obscured by the initial fluorescence peak, leading to increased uncertainty in τ
T . For SeNBD Die and SeNBD Glu in DMSO the τ
T values extracted were 1.4 μ
 s and 1.3 μ
 s, respectively. As the dynamics observed in the photosensitizer can be indicators of their efficacy, the established triplet-state lifetime information will feed back into further photosensitizer design and optimization.

**Fig. 4.**
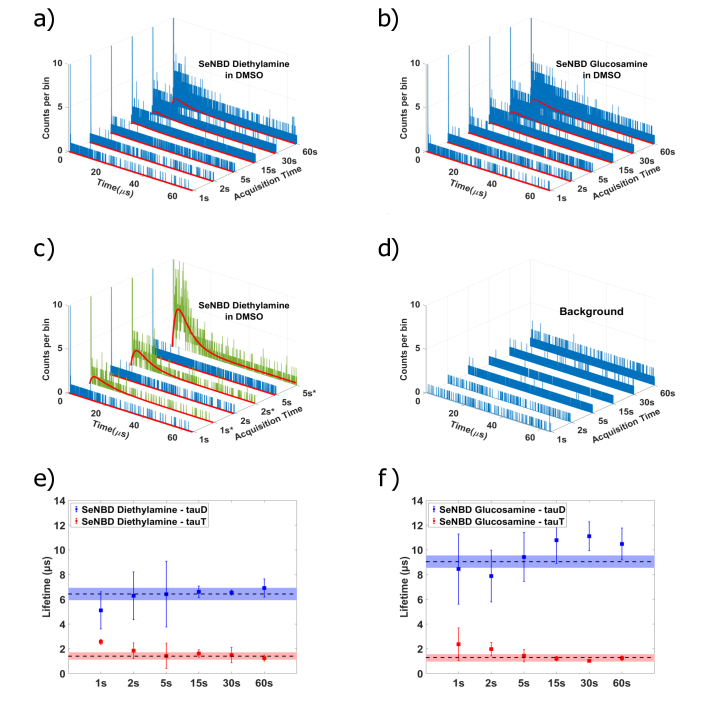
Rapidly acquired TCSPC histograms of photosensitized singlet-oxygen luminescence for SeNBD Die (a) and SeNBD Glu (b) in DMSO at full resolution of 1.024 ns/bin with data fits shown in red. (c) shows a comparison between the original full resolution histograms (in blue) and the same data with neighboring time bins summed to ∼
 65 ns per bin (in blue and marked with asterisk) for the 1 s, 2 s and 5 s acquisition times. See main text for details. (d) Shows the corresponding background measurement for each integration time. The time constants τ
D (blue) and τ
T (red) extracted from all rapid-acquisition-time measurements for SeNBD Die in DMSO and SeNBD Glu in DMSO are shown in (e) and (f), respectively. The dashed lines indicate the 30-minute mean value for their respective photosensitizer and solvent, whereas the shaded regions indicate the range of their reported uncertainty.

Having established the dynamics of the SeNBD-based photosensitizers over long signal acquisition times, we employ this highly sensitive single-photon detection system to detect photosensitized singlet-oxygen signatures with acquisition times as low as 1 second. Using this method we were able to provide rapid estimates of the τ
D and τ
T lifetimes from real-time measurements. Figure 4(a) and (b) show the TCSPC histograms obtained for SeNBD Die in DMSO and SeNBD Glu in DMSO for a range of acquisition times ≤
 60 seconds. At extremely short acquisition times (below 5 seconds), a phosphorescence signal curve is not immediately distinguishable in the TCSPC histograms. However, an increased density of populated time bins at early arrival times indicates the presence of the time-dependent phosphorescence signature. Alternatively, using a broader time bin in the TCSPC histogram visualizes the singlet-oxygen signal distribution more readily. This is illustrated in Fig. 4(c) which compares the 1 to 5 seconds acquisition time TCSPC histograms for two different bin sizes: the original 1.024 ns bin size shown in green, and a version of the same data produced using the summation of neighboring time bins to a total of ∼
 65 ns, shown in blue and identified by an asterisk. Using the minimization of residuals approach employed by a typical non-linear least squares fitting algorithm, even sub-5 second acquisition time TCSPC histograms could be fitted using Eq. (1). The lifetime values summarized in Fig. 4(e) and (f) are an average of the values extracted from fits to the short acquisition time TCSPC histograms (with the original bin size of 1.024 ns) of 3 measurements of 3 unique SeNBD Die in DMSO (e) and SeNBD Glu in DMSO (f) samples of the same make-up. The error bars shown are based on the standard deviation of results across the unique samples. To contextualize these short-acquisition-time results, dashed lines are included which indicate the τ
T and τ
D values obtained from the benchmark 30-minute acquisition-time measurements of the respective photosensitizer. Blue and red shaded areas indicate the range of long-time acquisition values (reported in Fig. 3) including their uncertainty. τ
D and τ
t lifetimes extracted from fits to the sub-60 second acquisition time measurements in SeNBD Die and SeNBD Glu in DMSO are consistent with the values established through long-time acquisition, even at extremely short acquisition times. Considering both photosensitizers and all lifetime values summarized in Fig. 4(e) and (f), we conclude that a high-accuracy lifetime determination could be carried out with acquisition times of at least 2 seconds. An acquisition time of 1 second still provided a reasonable indication of the longer lifetime, τ
D but determining τ
T is challenging. We note that summing neighboring time bins and fitting subsequently had little effect on the mean values of τ
T and τ
D , when the time bins were relatively short compared to the signal lifetimes. This demonstration of a rapid estimation of τ
D could be used to establish the presence of photosensitized singlet-oxygen and to identify a photosensitizer based on a known lifetime, in real-time.

## 4. Conclusion

Photosensitized singlet-oxygen luminescence signatures were extracted within seconds of acquisition time from novel SeNBD-based photosensitizers in solution using a low-noise, free-running InGaAs/InP SPAD detector in conjunction with the TCSPC method. Photosensitizer triplet-state and singlet-oxygen lifetimes were reliably extracted from TCSPC histograms acquired over only a few seconds, providing a methodology for real-time singlet-oxygen detection and photosensitizer identification. In a clinical setting, such as for PDT, the ability to detect singlet-oxygen production in short time frames would allow for real-time adjustments in light dosing, ensuring sufficient singlet-oxygen production and complete treatment of the diseased tissue. Although practical applications would introduce many other factors, by demonstrating the extraction of lifetimes in low-return signals this work represents a step towards the ability to make real-time adjustments using this type of singlet-oxygen detection technique. Future work would include further reducing this acquisition time for singlet-oxygen detection and lifetime identification. This could be achieved through a number of approaches: for example, by improving the optical throughput; use of higher detection efficiency SPADs; or by mitigating pulse-pile up [42,43].

## Data Availability

Data underlying the results presented in this paper are not publicly available at this time but may be obtained from the authors upon reasonable request.
